# Fostering Continuity of Care for Massachusetts Long-Term Care Residents on Medication for Opioid Use Disorder

**DOI:** 10.1093/geroni/igaa057.173

**Published:** 2020-12-16

**Authors:** Rosanna Bertrand, Chiara Moore, Katherine Fillo, Katherine Saunders, Stephanie Baker, Amy Berninger, Teresa Mota, Nicole Keane

**Affiliations:** 1 Abt Associates Inc., Cambridge, Massachusetts, United States; 2 MA Department of Public Health, Boston, Massachusetts, United States; 3 Healthcentric Advisors, Woburn, Massachusetts, United States; 4 Abt Associates, Inc., Cambridge, Massachusetts, United States

## Abstract

In 2016, the CDC estimated that 2.1 million Americans had Opioid Use Disorder (OUD); about 1.8 million related to prescribed painkillers. Older adults are especially susceptible; SAMHSA estimates that 2.7 million older adults will misuse prescription drugs by 2020. The Massachusetts Department of Public Health (MDPH) issued a 2016 Circular Letter advising long-term care facility (LTCF) administrators that, if otherwise eligible for admission, facilities are expected to admit individuals diagnosed with OUD, and provide medication for OUD (MOUD) as prescribed. Yet, many facilities express concern for admitting residents with OUD. The MDPH and their partners are conducting a multi-faceted training/technical support (TS) program to foster best practices across the continuum of care, targeting LTCF. The 15-month program consists of in-person learning sessions, a comprehensive toolkit, on-site TS, weekly contact, and a peer-to-peer webinar. Pre-training data indicated that 24 of 42 recruited LTCFs had not admitted residents with OUD. Although licensed LTCF practitioners can obtain a waiver to prescribe certain MOUD, only 4 of the 28 LTCF medical directors interviewed had done so. Subject matter experts led topic-specific discussions in the first learning session to educate on OUD/MOUD, dispel myths, make community connections, and provide resources. Almost all participants agreed that the session met the objectives of understanding OUD as a chronic disease, recognizing the stigma of OUD, gaining knowledge of MOUD treatments, and obtaining strategies to enhance best practices across the continuum of care. All items on the pre/post-session assessments indicated a significant increase in understanding (37% versus 60%, respectively).

